# Clinical and epidemiological characterization of patients with cutaneous tuberculosis treated at a referral center in the Brazilian Amazon: case series^☆^

**DOI:** 10.1016/j.abd.2024.05.007

**Published:** 2025-01-14

**Authors:** Letícia Rezende da Silva Sobral, Isabela de Nazaré Tavares Cardoso Souza, Maria Fernanda de Almeida Cavalcante Aranha, Ana Beatriz Dias Silva, Maria Amélia Lopes dos Santos, Renata Mie Oyama Okajima, Francisca Regina Oliveira Carneiro, Carla Andréa Avelar Pires

**Affiliations:** a Dermatology Service, Universidade do Estado do Pará, Belém, PA, Brazil; b Faculty of Medicine, Universidade do Estado do Pará, Belém, PA, Brazil

**Keywords:** Demography, Epidemiology, Tuberculosis, Cutaneous, Tuberculosis, Extrapulmonary

## Abstract

**Background:**

Cutaneous tuberculosis is a rare form of the disease that defies diagnosis due to the diversity of clinical presentations. This study was based on the reality of a dermatology referral center in the Brazilian Amazon region to detail several characteristics of this disease.

**Objective:**

To describe a series of cases of cutaneous tuberculosis treated at a dermatology service in the Brazilian Amazon region, addressing epidemiological aspects, clinical forms, diagnostic methods, treatment, and outcomes.

**Methods:**

This is a descriptive and observational study of the case series type, including eight patients with a confirmed diagnosis of cutaneous tuberculosis seen between 2021 and 2023. A standardized protocol was used to collect data from patients' medical records.

**Results:**

Among the eight cases of cutaneous tuberculosis, there were four cases of erythema induratum of Bazin, one case of tuberculosis verrucosa cutis, two cases of scrofuloderma, and one case of lupus vulgaris. Erythema induratum of Bazin, the most common form in the study, occurred predominantly in women, presenting as infiltrated plaques and erythematous nodules. All eight cases were treated with the standard therapeutic regimen of the Brazilian Ministry of Health (rifampicin, isoniazid, pyrazinamide, and ethambutol).

**Study limitations:**

They include the small sample size, making generalizations difficult.

**Conclusions:**

The study addressed the clinical diversity of cutaneous tuberculosis, with erythema induratum of Bazin as the most common form. The importance of the socioeconomic context in the prevalence of the disease and the need for more comprehensive epidemiological studies to improve the understanding of cutaneous tuberculosis, especially in endemic regions, are highlighted.

## Introduction

Tuberculosis (TB) is characterized as an infectious disease that can be caused by a group of seven mycobacteria that constitute the *Mycobacterium tuberculosis* complex, namely *M. tuberculosis*, *M. bovis*, *M. africanum*, *M. canetti*, *M. microti*, *M. pinnipedii,* and *M. caprae*.^1^ *Mycobacterium tuberculosis*, also called Koch bacillus, is undoubtedly the most important etiological agent of tuberculosis since it is responsible for the majority of cases.^1^

Despite being a preventable, treatable, and curable disease, TB is still among the ten leading causes of death worldwide. In Brazil, tuberculosis is one of the main infectious diseases in regions lacking economic resources, associated with social exclusion and the HIV-infection, in addition to being more prevalent in males (66.5%). In 2019, 73,684 new cases of tuberculosis were diagnosed in Brazil, with an incidence of 35 cases/100,000 inhabitants and 4,490 deaths due to the disease. Approximately 14% of the cases are extrapulmonary; of these, only 1% to 2% are reported with cutaneous involvement. When stratified by federation unit, it is evident that the highest incidence rates, above 51 cases/100,000 inhabitants, is found in the state of Rio de Janeiro, located in the southeastern region of the country and in a large part of the Brazilian Amazon region, including the states of Amazonas, Pará, Roraima, and Acre.^2^, ^3^, ^4^

From a regional perspective, from 2018 to 2022, there was a linear increase in the tuberculosis incidence rate in the state of Pará, increasing from 47.95 cases/100,000 inhabitants in 2018 to 55.14 cases/100,000 inhabitants in 2022, with an average of 49 cases/100,000 inhabitants. In 2020, there was a reduction in these values, which increased again in 2021. Regarding the extrapulmonary forms, in children under five years of age, there was a variation in the historical series, with 24.6% of new TB cases presenting the extrapulmonary clinical form in 2018, a proportion that reached its highest percentage in 2020 (25.8%), with a slight decrease in 2021 and 2022, reaching 21.2% and 11.6%, respectively.^5^

The BCG (Bacillus Calmette-Guérin) vaccination coverage in the state of Pará, considering the years 2018 to 2021, was the third lowest in the country, with an average of 78.87%, behind only the states of Bahia (76.90%) and Rio de Janeiro (78.78%). Throughout this period, there was a progressive reduction in this coverage, both in the state of Pará and nationwide,^6^ making the population more susceptible to the disease.

Tuberculosis can present itself in two clinical forms, extrapulmonary and pulmonary, with the latter being the most common and statistically important. However, the extrapulmonary forms are more diverse since *M. tuberculosis* can reach many tissues and organs in the body. Among the main forms of extrapulmonary tuberculosis, bone, lymph node, pericardial, and cutaneous tuberculosis (CTB) stand out, with the latter being the most uncommon, corresponding to 1% to 2% of cases.^7^, ^8^, ^9^

Cutaneous tuberculosis was first described in the literature in the 19^th^ century, and even today, it is considered rare, and its physiological mechanism is little known. Nevertheless, like most other forms of presentation, cutaneous tuberculosis is also caused mainly by *Mycobacterium tuberculosis*, with some exceptions in which the cause is *M. bovis* or the Bacillus Calmette-Guérin (BCG) vaccine. The clinical picture of CTB is considered complex, as it shows lesions that mimic several other cutaneous diseases, which can present clinically as verrucous plaques, suppurative nodules, inflammatory papules, chronic ulcers, and others, making the diagnosis a challenge.^10^

CTB can be classified according to its form of inoculation, as exogenous cutaneous tuberculosis, in which the clinical presentations are tuberculous chancre and tuberculosis verrucosa cutis; and endogenous cutaneous tuberculosis, with its main clinical presentations being scrofuloderma, lupus vulgaris, orificial tuberculosis, tuberculous gumma, and acute miliary tuberculosis. In addition, cutaneous tuberculosis can also occur as hypersensitivity reactions, called tuberculids, with emphasis on lichen scrofulosorum, erythema induratum of Bazin, and papulonecrotic tuberculid.^8^, ^10^, ^11^, ^12^

Although there are tests with great sensitivity for the diagnosis of CTB, such as the automated and non-radiometric system for the culture of mycobacteria, in practice, the management follows the same principles as for the pulmonary form, with direct microscopic examination or bacilloscopy being commonly performed, in search of AFB (acid-fast bacilli), using the Ziehl-Neelsen staining method. Although it is the most widely used method, when it comes to cutaneous tuberculosis, the sensitivity of bacilloscopy is less than 10%.^10^

Until 2009, TB treatment was carried out only with a combination of rifampicin (R), isoniazid (H) and pyrazinamide (Z). However, the Ministry of Health (MoH), through the National TB Control Program, associated ethambutol (E) antituberculosis multidrug therapy (MDT) protocol. Currently, in Brazil, the basic treatment regimen for all forms of TB is carried out with the association of RHZE in the first two months of treatment (intensive phase), followed by another four months of RH (maintenance phase). Only two exceptions are established, the first being for patients with meningoencephalic and/or osteoarticular TB, in which treatment must be carried out for ten months in the maintenance phase, totaling 12 months of treatment, and the second for children under ten years of age, in whom ethambutol cannot be used due to the high risk of ocular damage.^13^, ^14^, ^15^

Although rare, cutaneous tuberculosis has several clinical forms, which makes its diagnosis difficult due to the lack of studies on the subject. Therefore, the present study aims to describe a series of cases with information on the epidemiological profile, clinical presentations, histopathological findings, diagnosis, treatment, and its adverse effects on patients with TB treated at a secondary referral center in Dermatology in the Brazilian Amazon region.

## Methods

This is a descriptive, observational, and single-center study of the case series type carried out at a Dermatology Service that is a secondary care referral service in the treatment of general and tropical dermatological diseases in the region. The research was carried out after approval by the Research Ethics Committee of the University, under Counsel number 6,277,142/2023, and after authorization by the Dermatology Service coordination. It is also worth mentioning that the present study followed the CARE checklist for case reports and series.

A convenience sample was used, consisting of patients treated in the years 2021 to 2023 at the aforementioned service, with confirmed clinical and histopathological diagnoses of cutaneous tuberculosis. The culture tests for mycobacteria were performed using the Lowenstein medium.

A standardized protocol developed by the researchers was used to collect data from the medical records. The considered variables were epidemiological data, clinical and histopathological aspects of the lesions, diagnostic methods, type of treatment, and clinical outcomes.

## Results

Eight cases of cutaneous tuberculosis were identified during the study period. Among them, four were erythema induratum of Bazin, one was tuberculosis verrucosa cutis, two cases were scrofuloderma, and one was lupus vulgaris. Their clinical and epidemiological profiles are described in Table 1.

**Table 1:** Clinical and epidemiological profile of cutaneous tuberculosis cases treated at a referral center from 2021 to 2023.

Case	Subtype	Age	Gender	Area of Residence	Occupation	Level of schooling	PPD test	BCG scar	Previous contact with TB
1	Erythema induratum of Bazin	57 years	Female	Ananindeua-PA	Homemaker	Incomplete Elementary School	12 mm	Yes	Yes
2	Erythema induratum of Bazin	53 years	Female	Santa Bárbara-PA	Homemaker	Incomplete Elementary School	19 mm	Yes	Yes
3	Erythema induratum of Bazin	9 years	Female	Soure-PA	Student	Incomplete Elementary School	13 mm	Yes	Yes
4	Erythema induratum of Bazin	17 years	Male	Ananindeua-PA	Student	Incomplete High School	20 mm	Yes	No
5	TB verrucosa cutis	36 years	Male	Reservation near the region of Gurupi-MA	Agricultural worker	Illiterate	18 mm	Yes	Yes
6	Scrofuloderma	49 years	Male	Tracuateua-PA	Agricultural worker	Incomplete Elementary School	15 mm	No	No
7	Lupus vulgaris	25 years	Female	Santo Antônio do Tauá-PA	Agricultural worker	–	14 mm	Yes	Yes
8	Scrofuloderma	40 years	Male	Igarapé-Açu-PA	Designer	Complete High School	20 mm	Yes	No

PA, state of Pará; MA, state of Maranhão.

### Erythema induratum of Bazin

**Fig. 1:**
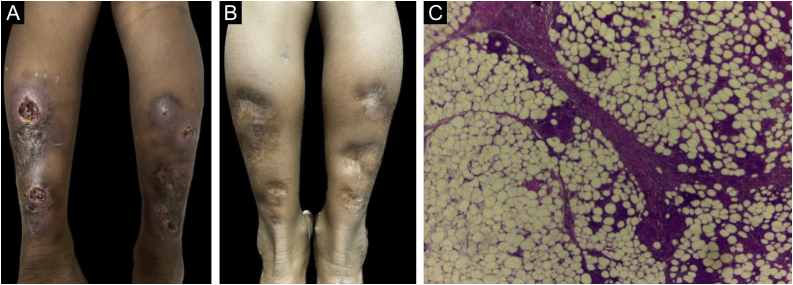
(A) Erythema induratum of Bazin – ulcerated nodules, topped by a serohematic crust and atrophic plaque, on the posterior region of the legs. (B) Erythema induratum of Bazin – post-treatment lesions. (C) Erythema induratum of Bazin – septal and lobular granulomatous panniculitis (Hematoxylin & eosin, ×400).

Among the four cases of erythema induratum of Bazin, three were in females and one in male, with ages ranging from nine to 57 years. Patients presented erythematous and brownish infiltrated plaques and painful erythematous nodules, mostly located on the calves and lateral and medial regions of the leg, of long-term evolution from two to nine years. Some lesions developed into ulcers and were associated with edema and pruritus (Fig. 1A). Only one of the patients denied contact with people who had treated or were under treatment for tuberculosis.

Skin and sputum bacilloscopy, culture, purified protein derivative (PPD) test, histopathology, EAS urine test, chest X-ray, and computed tomography (CT) of the chest and abdomen were performed. Bacilloscopies and cultures showed absence of AFB in the assessed samples. The EAS test and chest X-ray showed no changes, corroborating the findings of the chest and abdominal CT scans. PPD values ​​ranged from 12 to 20 mm.

RHZE was used for the treatment, with mild adverse effects, in all cases of erythema induratum of Bazin. No patient required repeated treatment and all showed apparent cure.

It is worth mentioning the nine-year-old patient, from a rural area, with non-painful plaques and nodules that had developed into ulcers on her calves three years before, with a previous history of contact at home with active pulmonary tuberculosis at the age of three. The dermatological examination revealed infiltrated erythematous plaques, with two ulcerated nodules and atrophic plaques in the posterior region of the lower limbs (Fig. 1A). Histopathology revealed a dense and diffuse granulomatous inflammatory infiltrate in the dermis and septa and lobules of the subcutaneous tissue, with lymphocytes, histiocytes, epithelioid cells, and multinucleated Langhans giant cells. There were also caseous necrosis foci. Fite-Faraco staining did not disclose AFB (Fig. 1C) and the PPD measured 13 mm. Chest X-ray, laboratory tests, and urine testing showed no changes. The patient was treated with the RHZ regimen, as recommended by the Ministry of Health according to the weight (25 kg), with a dose of 300, 200, and 750 mg/day, respectively. One month after starting treatment, there was significant improvement in the lesions, progressing to apparent cure (Fig. 1B). The patient is under monitoring and has cicatricial lesions, with no signs of active disease.

### Tuberculosis verrucosa cutis

**Fig. 2:**
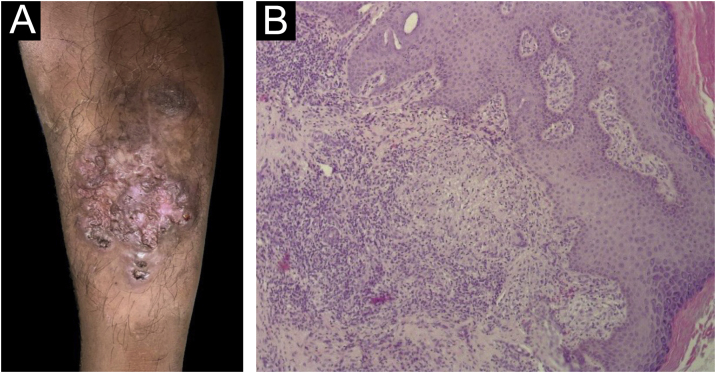
(A) Tuberculosis verrucosa cutis – erythematous vegetative plaque with verrucous surface and atrophic central region in the anterior region of the left leg. (B) Tuberculosis verrucosa cutis – hyperplastic epidermis and epithelioid granulomas with Langhans giant cells in the dermis (Hematoxylin & eosin, ×100).

Regarding the only case of tuberculosis verrucosa cutis, the patient was a 36-year-old indigenous male who lives in a reservation near the Gurupi region, in the state of Maranhão. The lesion appeared after local trauma caused by a thorn three years before, with progressive growth, associated with erythema, pruritus and local pain. On examination, an erythematous vegetative plaque with a verrucous surface and an atrophic central region was observed, measuring 10 cm, as well as a normochromic nodule on the periphery of the plaque, located in the anterior region of the left leg (Fig. 2A). There had been previous contact with a patient diagnosed with pulmonary tuberculosis.

Skin bacilloscopy and culture showed the absence of AFB in the sample; histopathology and PCR tests for *Leishmania* and the direct mycological examination of the lesion for fungi showed negative results. The PPD measured 18 mm. Histopathology disclosed pseudoepitheliomatous epithelial hyperplasia and in the dermis a mixed inflammatory infiltrate with histiocytes, lymphocytes, plasma cells, mast cells, epithelioid cells, multinucleated Langhans giant cells, neutrophils in the suppuration areas, and epithelioid cell granulomas, some with central suppurative necrosis. The infiltrate extended to the deep reticular dermis (Fig. 2B).

The patient has received treatment and progressed with clinical improvement and total regression of the lesions depicted in Fig. 2A. There have been no reports of treatment abandonment/irregular use to date, but there have been reports of adverse effects, such as nausea and vomiting.

### Scrofuloderma

**Fig. 3:**
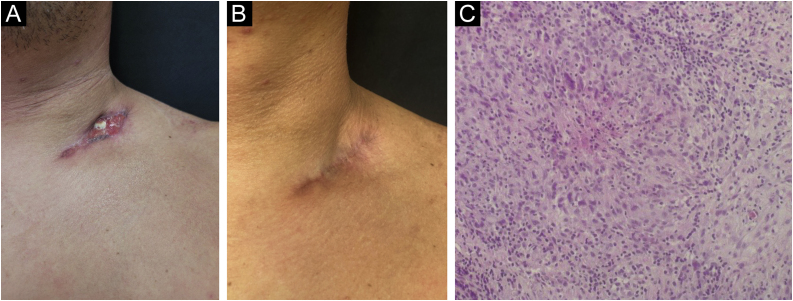
(A) Scrofuloderma – ulceration in the left supraclavicular region. (B) Scrofuloderma – post-treatment lesion. (C) Scrofuloderma – epithelioid granuloma with central necrosis and multinucleated Langhans giant cells (Hematoxylin & eosin, ×200).

The two cases of scrofuloderma presented in males, aged 40 and 49, with a history of nodular lesions of progressive growth, which developed ulceration and secretion. On examination, a single ulceration with violaceous edges was observed, showing background granulation tissue on an erythematous base measuring 2.5 cm by 3 cm, with clear borders and regular contours, located in the left supraclavicular region (Fig. 3A); and a hardened normochromic nodule, more palpable than visible, located in the proximal medial region of the left thigh. The patients denied contact with individuals who had treated or were under treatment for tuberculosis.

PPD and histopathology were performed, as well as skin bacilloscopy, which disclosed absence of AFB; culture and chest and abdominal CT scans revealed no alterations. PPD tests measured 15 and 20 mm. The 40-year-old patient, with a PPD of 20 mm, had a positive culture for *Mycobacterium tuberculosis*. Histopathology showed an ulcerated epidermis and abundant mixed inflammatory infiltrate with neutrophils, lymphocytes, and histiocytes. In the dermis, small epithelioid granulomas with central necrosis, and multinucleated giant cells, foreign body and Langhans types, were observed (Fig. 3C).

Both patients underwent the standard tuberculosis treatment regimen and are under monitoring, showing apparent cure, as shown in Fig. 3B, and without lesion recurrence.

### Lupus vulgaris

**Fig. 4:**
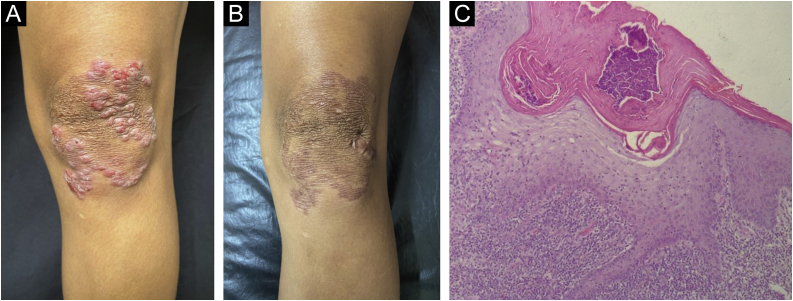
(A) Lupus vulgaris – erythematous-violaceous, infiltrated plaque with keloid-like surface on the left knee. (B) Lupus vulgaris – post-treatment lesion. (C) Lupus vulgaris – epidermis hyperplasia, crust on the surface, abundant subepidermal inflammatory infiltrate, with epithelioid granuloma, without necrosis (Hematoxylin & eosin, ×100).

The lupus vulgaris subtype was identified in a female patient, aged 25 years, with lesions that had begun after a local injury, with progressive evolution, bleeding, and drainage of purulent secretion for ten years. She denied contact with patients who had been treated for tuberculosis. On dermatological examination, an erythematous-violaceous, infiltrated plaque with a keloid-like surface, with clear limits and irregular contours, measuring 7 × 6 cm, was observed on the left knee (Fig. 4A). It showed an “apple jelly” appearance on the diascopy.

Skin and sputum bacilloscopy and culture showed absence of AFB in the assessed samples. Chest CT showed a non-calcified non-specific nodule and abdominal CT showed no alterations. The PPD test measured 14 mm. Histopathology showed a partially ulcerated epidermis with irregular acanthosis. In the superficial and deep dermis, there was a mixed inflammatory infiltrate containing lymphocytes, macrophages, neutrophils, eosinophils, and tuberculoid granulomas with multinucleated Langhans giant cells, without necrosis (Fig. 4C).

Treatment was carried out for six months with an effective outcome (Fig. 4B) and no adverse effects.

## Discussion

In endemic areas, including Brazil, the percentage of CTB represents 0.1% to 2% of dermatological complaints,^16^, ^17^, ^18^, ^19^ in contrast to other regions with low prevalence, where the values ​​do not exceed 0.06%.^20^, ^21^, ^22^ This scenario is related to socioeconomic issues, since poverty, low level of schooling, poor nutrition, poor sanitary conditions, and inadequate access to healthcare are conditions associated with the spread of TB, worse prognosis, and inadequate treatment.^23^, ^24^, ^25^ The state of Pará, where the present study took place, presented an alarming situation in 2021, occupying, the 23^rd^ position among the Human Development Indexes (HDI) of Brazilian states, with a value of 0.69.^26^ Despite the scarcity of data on TB in the state of Pará, it is likely that socioeconomic issues and the mean HDI are also responsible for the spread of the disease.

Only 5% to 10% of patients who have had contact with infected individuals will have active tuberculosis. The main form of transmission occurs through inhalation of droplets or aerosol, but it can also occur by ingestion or inoculation through a break in the mucocutaneous barrier that can facilitate the entry of the bacillus. Moreover, transmission can occur by contiguity, even in the presence of intact skin, generating exogenous forms of cutaneous and/or mucous membrane tuberculosis.^12^ All patients in the study were investigated for systemic disease, but none showed active symptoms or suggestive symptoms of pulmonary disease or any other systemic focus.

The clinical-histopathological manifestations, onset, and progression of CTB depend on a complex interaction between bacterial virulence, the patients previous contact with the bacillus, the hosts cellular immunity, the anatomical location, and the route of inoculation,^8^ thus resulting in polymorphism of the lesions.^11^

CTB can be classified as primary (absence of previous contact with the bacillus) and secondary (in a previously sensitized patient). The infection can occur exogenously when inoculation occurs directly into the skin, or endogenously, in which skin involvement is a consequence of a distant primary focus of active tuberculosis, resulting from autoinoculation, contiguity or dissemination by the hematogenous route. Depending on the degree of specific hypersensitivity, patients can also be classified as having cutaneous tuberculosis and tuberculids.^3^

Exogenous cutaneous tuberculosis manifests as a tuberculous chancre in individuals who have not had previous contact with the tuberculosis bacillus, or in a previously infected or sensitized patient, as tuberculosis verrucosa cutis (TBVC).^27^, ^28^ TBVC was present in one of the cases in the study, in which the patient was previously vaccinated and had previous contact with the bacillus.

In endogenous forms, when there is autoinoculation in the mucosa and periorificial skin in individuals with low immunity who have tuberculosis in internal organs, orificial tuberculosis may occur. Through hematogenous spread, a host with compromised immunity may develop acute miliary tuberculosis, while in a host with some immunity to tuberculosis, this spread may result in lupus vulgaris,^29^, ^30^ as occurred in a case reported in the present study, in a patient who received immunization and had previous contact with tuberculosis. Contiguous spread from lymph nodes or bones results in the form known as scrofuloderma,^27^ which affected two of the studied patients.

In tuberculids, the inoculation route is endogenous. Most recent studies do not consider tuberculids to be authentic forms of CTB, but rather a heterogeneous group of clinical entities that result from the hosts hypersensitivity response to the bacillus. Bacilloscopy is usually negative for AFB in skin lesions. One of the tuberculids is erythema induratum of Bazin, characterized by subcutaneous, chronic and recurrent, symmetrical and painless erythematous-violaceous nodules and plaques, preferably located on the back of the legs, but can also affect other sites, more frequently in women,^3^ corroborating the data of the present study.

There is a downward trend in the occurrence of endogenous and exogenous forms of CTB, mainly scrofuloderma and TBVC, and an increase in tuberculids, especially erythema induratum of Bazin and papulonecrotic tuberculids.^21^, ^22^ In the Dermatology referral service where the study was conducted, erythema induratum of Bazin accounted for half of the analyzed cases.

In addition, although TB ​​can affect people of any age and gender, in Brazil TB is more prevalent in women,children, and young adults,^17^, ^31^, ^32^ as in other developing countries.^16^, ^33^ In the present study, there was an equal distribution in the genders and heterogeneity in relation to age, a situation that may be related to the small number of patients, not necessarily reflecting the local reality.

Regarding the diagnostic methods for CTB, isolation in culture is the reference,^34^ ensuring, in addition to the definitive diagnosis, the susceptibility testing of drugs used in the treatment.^35^ Despite its high specificity, the culture is a method with low sensitivity, due to the scarcity of bacilli in skin lesions, in addition to the time required for attaining the result, which can take up to three weeks in liquid medium and eight weeks in solid medium.^34^, ^35^

Therefore, it is observed in the present study, as in others, that culture, despite being a test that is always requested and that when positive confirms the diagnosis, has an extremely high negative rate. Thus, reiterating what was identified in the literature, it was observed that only one of the eight cases of CTB had a positive culture, also explaining why this test is not mandatory to establish the diagnosis.^36^, ^37^

The number of bacilli also interferes with detecting through AFB staining, which differs according to the type of cutaneous TB, ranging from numerous bacilli in TB chancre to none in tuberculids in bacilloscopy.^14^ By presenting only the clinical forms of erythema induratum of Bazin, TBVC, scrofuloderma, and LV, this series of cases attained 100% negative bacilloscopy results. It is important to highlight that, in this study, only one case (scrofuloderma) undoubtedly met the diagnostic criteria, with a positive culture, while the other cases had their diagnosis supported by clinical and histopathological findings, PPD tests, and response to a specific treatment.

Regarding the histopathological aspects on Hematoxylin & eosin stained slides, CTB shows as characteristics the presence of caseation necrosis, giant cells, and epithelioid granulomas.^14^ It is worth noting that clinical variants of the disease can also be identified on histopathology, since TBVC shows prominent hyperkeratosis with pseudoepitheliomatous hyperplasia and neutrophilic abscesses in the papillary dermis; lupus vulgaris shows epidermal changes that vary from hyperkeratosis to acanthosis or epidermal thinning and, generally, loosely formed tuberculoid granulomas or a confluent granulomatous infiltrate located in the papillary dermis, although well-formed granulomas are present to varying degrees.^14^

Moreover, all cases of scrofuloderma show tuberculoid granulomas located mainly in the reticular dermis, and neutrophilic abscesses are observed in most patients.^14^ In turn, erythema induratum of Bazin shows panniculitis, lymphocytic vasculitis, and areas of fat necrosis.^14^ Based on this, it was observed that the present study obtained results similar to those described in the available literature.

Furthermore, the tuberculin test performed by intradermal injection of 5 tuberculin units of purified protein derivatives (PPD) derived from the attenuated strain of *Mycobacterium tuberculosis* is used to diagnose latent TB and as a complement in the diagnosis of active TB;^15^ however, it has low sensitivity and specificity for TB, due to the variable immunological spectrum among the different clinical types of the disease.^38^ Based on this, the tuberculin test may be negative in early tuberculous chancre, disseminated miliary tuberculosis, and orificial tuberculosis, although it is generally strongly positive in LV, scrofuloderma, TBVC, and tuberculids,^39^ which corroborates the findings of this case series, in which PPD test of different values were seen in all cases.

Finally, although they were not requested in the present study due to their limited availability in the Brazilian Unified Health System (SUS, *Sistema Único de Saúde*) in the state of Pará, the rapid molecular test for tuberculosis (RMT-TB) and the *in vitro* interferon-gamma release assay, also called Quantiferon-TB (IGRA), are extremely useful tests in the evaluation of such patients. RMT-TB is based on real-time polymerase chain reaction (RT-PCR) and is currently considered the first-choice test to establish TB diagnosis, both for its high sensitivity and specificity and for directly showing the resistance of these microorganisms to rifampicin. However, it should not be used for patient monitoring, as its positivity persists, since this technique identifies the DNA of mycobacteria, whether they are viable or not.^14^

IGRA is based on the principle that when a patient is exposed to TB antigens, it is possible to identify the *in vitro* levels of interferon-gamma produced by the stimulated T cells. Although it is a more recent test for identifying latent TB, it has some advantages over the PPD test, such as the absence of interference from the BCG vaccine. However, to date, there is no established superiority of any of these tests.^40^

It is noteworthy that the request for imaging tests, such as chest and abdominal CT, chest X-ray, and lymph node ultrasound, as well as EAS, for patients with TB in the health service studied, was in accordance with current literature. This is due to the fact that they are screening methods for all TB cases in search of manifestations of extrapulmonary TB.^14^

As previously mentioned, the standardized treatment for TB by the Brazilian Ministry of Health, with a few exceptions, is the MDT, associating RHZE for two months, followed by another four months of RH. All CTB patients treated at this referral center in the Brazilian Amazon region were managed with the same treatment implemented for other forms of extrapulmonary TB.^10^ The eight patients are still under monitoring, with all of them apparently cured after treatment , showing improvement of the lesions.

Adverse reactions related to anti-tuberculosis MDT are well-known in the literature since they involve a wide range of symptoms. Among the main adverse effects observed in patients at this referral center, the following stand out: nausea, vomiting, epigastric burning, change in urine color, and paresthesia of the upper and lower limbs, which is consistent with most of the symptoms observed in the studies, including neurological, articular, hepatic and, mainly, gastrointestinal symptoms. In some patients, it is necessary to replace or temporarily discontinue the first-line drugs for TB management due to intolerable adverse effects, such as a great increase in aminotransferase levels,^10^ which was not necessary for the patients treated in this study.

## Conclusion

Despite the limitations inherent to case series, this study reinforces the need for greater attention and additional studies on the presentation of tuberculosis, especially in endemic regions, aiming to improve early diagnosis and adequate treatment focusing in a better prognosis. It is also suggested that larger-scale epidemiological studies on CTB be developed, as there is a scarcity of data on the subject. This hinders the understanding of the scenario related to the disease in Brazil and specially in Pará, thus, impeding the better management of the disease.

## Financial support

This study was funded by the 10.13039/501100003593National Council for Scientific and Technological Development (CNPq, *Conselho Nacional de Desenvolvimento Científico e Tecnológico*) and the Amazon Foundation for Support of Studies and Research of Pará (FAPESPA, *Fundação Amazônia de Amparo a Estudos e Pesquisas*) through the Institutional Program for Undergraduate Research Grants (PIBIC, *Programa Institucional de Bolsas de Iniciação Científica*) number 026/2023 – Universidade do Estado do Pará (UEPA).

## Authors’ contributions

Letícia Rezende da Silva Sobral: Collection of data, or analysis and interpretation of data; statistical analysis; drafting and editing of the manuscript or critical review of important intellectual content; collection, analysis and interpretation of data; critical review of the literature; approval of the final version of the manuscript.

Isabela de Nazaré Tavares Cardoso Souza: Collection of data, or analysis and interpretation of data; statistical analysis; drafting and editing of the manuscript or critical review of important intellectual content; collection, analysis and interpretation of data; critical review of the literature; approval of the final version of the manuscript.

Maria Fernanda de Almeida Cavalcante Aranha: Collection of data, or analysis and interpretation of data; statistical analysis; drafting and editing of the manuscript or critical review of important intellectual content; collection, analysis and interpretation of data; critical review of the literature; approval of the final version of the manuscript.

Ana Beatriz Dias Silva: Collection of data, or analysis and interpretation of data; statistical analysis; drafting and editing of the manuscript or critical review of important intellectual content; collection, analysis and interpretation of data; critical review of the literature; approval of the final version of the manuscript.

Maria Amélia Lopes dos Santos: Design and planning of the study; effective participation in research orientation; intellectual participation in the propaedeutic and/or therapeutic conduct of the studied cases; critical review of the literature; approval of the final version of the manuscript.

Renata Mie Oyama Okajima: Design and planning of the study; effective participation in research orientation; intellectual participation in the propaedeutic and/or therapeutic conduct of the studied cases; critical review of the literature; approval of the final version of the manuscript.

Francisca Regina Oliveira Carneiro: Design and planning of the study; effective participation in research orientation; intellectual participation in the propaedeutic and/or therapeutic conduct of the studied cases; critical review of the literature; approval of the final version of the manuscript.

Carla Andrea Avelar Pires: Design and planning of the study; effective participation in research orientation; intellectual participation in the propaedeutic and/or therapeutic conduct of the studied cases; critical review of the literature; approval of the final version of the manuscript.

## Conflicts of interest

None declared.

## References

[1] Santos CRB. Tuberculose miliar em paciente imunocompetente: relato de caso e revisão da literatura [graduation thesis]. Hospital do Servidor Público Municipal, 2022. Available from: https://docs.bvsalud.org/biblioref/2022/05/1370024/tcc-cecilia-santos.pdf.

[2] Oliveira G.C.A., Silva A.C.S.S., Regazzi I.C.R., Nasser M.R.M., Brust R.S., Knupp V.M.A.O. (2021). Epidemiological profile of the population with tuberculosis in the Rio de Janeiro State. Rev Pesqui Cuid é Fundam Online.

[3] Azulay R.D., Azulay D.R., Azulay-Abulafia L. (2022).

[4] Hernández-Solís A., Quintana-Martínez A., Quintanar-Ramírez M.I., Álvarez-Maldonado P., Reding-Bernal A. (2023). Tuberculosis extrapulmonar: un problema de salud pública. Cir Cir.

[5] Secretaria de Saúde Pública do Estado do Pará (2023). Boletim Epidemiológico Tuberculose 2023.

[6] Ministério da Saúde. BCG por Ano segundo Unidade da Federação [Internet]. DATASUS – TABNET; Available from: https://datasus.saude.gov.br/informacoes-de-saude-tabnet/.

[7] Kaul S., Kaur I., Mehta S., Singal A. (2023). Cutaneous tuberculosis. Part I: pathogenesis, classification, and clinical features. J Am Acad Dermatol.

[8] De Vita E., Segala F.V., Amone J., Samuel K., Marotta C., Putoto G. (2022). Subacute cardiac tamponade due to tuberculous pericarditis diagnosed by urine lipoarabinomannan assay in an immunocompetent patient in Oyam district, Uganda: a case report. Int J Environ Res Public Health.

[9] Santos J.B., Figueiredo A.R., Ferraz C.E., Oliveira M.H., Silva P.G., Medeiros V.L.S. (2014). Cutaneous tuberculosis: epidemiologic, etiopathogenic and clinical aspects ‒ Part I. An Bras Dermatol..

[10] Mann D. Evolução clínica e terapêutica dos pacientes com tuberculose cutânea atendidos no Instituto Nacional de Infectologia Evandro Chagas, Fiocruz - RJ, entre 2000 e 2016 [dissertation]. Fundação Oswaldo Cruz. Instituto Nacional de Infectologia Evandro Chagas, 2018. Available from: https://www.arca.fiocruz.br/bitstream/handle/icict/37299/danielle_mann_ini_mest_2018.pdf?sequence=2&isAllowed=y.

[11] Brito A.C., Oliveira C.M.M., Unger D.A.-A., Bittencourt M.J.S. (2022). Cutaneous tuberculosis: epidemiological, clinical, diagnostic and therapeutic update. An Bras Dermatol.

[12] Santos P.F.A.M., Condino-Neto A., Gomes L.N., Cardoso C.A. (2022). Intrathoracic tuberculosis in the pseudotumoral and bone form as a manifestation of chronic granulomatous disease. Arq Asmas Alerg Imunol.

[13] Secretaria de Estado de Saúde do Rio de Janeiro (2023). Guia para Controle de Tuberculose em Instituições de Acolhimento para População em Situação de Rua.

[14] Kaul S., Jakhar D., Mehta S., Singal A. (2023). Cutaneous tuberculosis. Part II: complications, diagnostic workup, histopathologic features, and treatment. J Am Acad Dermatol.

[15] Lee E., Holzman R.S. (2002). Evolution and current use of the tuberculin test. Clin Infect Dis.

[16] Pandhi D., Reddy B.S.N., Chowdhary S., Khurana N. (2004). Cutaneous tuberculosis in Indian children: the importance of screening for involvement of internal organs. J Eur Acad Dermatol Venereol.

[17] Spelta K., Diniz L.M. (2016). Cutaneous tuberculosis: a 26-year retrospective study in an endemic area of tuberculosis, Vitoria, Espırito Santo. Brazil Rev Inst Med Trop Sao Paulo.

[18] Kumar B., Muralidhar S. (1999). Cutaneous tuberculosis: a twenty-year prospective study. Int J Tuberc Lung Dis.

[19] Zouhair K., Akhdari N., Nejjam F., Ouazzani T., Lakhdar H. (2007). Cutaneous tuberculosis in morocco. Int J Infect Dis.

[20] Hamada M., Urabe K., Moroi Y., Miyazaki M., Furue M. (2002). Epidemiology of cutaneous tuberculosis in Japan: a retrospective study from 1906 to 2002. Int J Dermatol.

[21] Ho C.K., Ho M.H., Chong L.Y. (2006). Cutaneous tuberculosis in Hong Kong: an update. Hong Kong Med J.

[22] Chong L.Y., Lo K.K. (1995). Cutaneous tuberculosis in Hong Kong: a 10-year retrospective study. Int J Dermatol.

[23] Marimani M., Ahmad A., Duse A. (2018). The role of epigenetics, bacterial and host factors in progression of Mycobacterium tuberculosis infection. Tuberculosis (Edinb).

[24] Muniyandi M., Ramachandran R. (2008). Socioeconomic inequalities of tuberculosis in India. Expert Opin Pharmacother.

[25] Wang Q., Guo L., Wang J., Zhang L., Zhu W., Yuan Y. (2019). Spatial distribution of tuberculosis and its socioeconomic influencing factors in mainland China 2013–2016. Trop Med Int Health.

[26] Instituto Brasileiro de Geografia e Estatística (IBGE) (2021). Índice de Desenvolvimento Humano por Unidade da Federação.

[27] Sehgal V.N., Bhattacharya S.N., Jain S., Logani K. (1994). Cutaneous tuberculosis: the evolving scenario. Int J Dermatol.

[28] Dhawan A.K., Pandhi D., Wadhwa N., Singal A. (2015). Tattoo inoculation lupus vulgaris in two brothers. Indian J Dermatol Venereol Leprol.

[29] Dias M.F.R.G., Bernardes Filho F., Quaresma M.V., Nascimento L.V., Nery J.A.C., Azulay D.R. (2014). Update on cutaneous tuberculosis. An Bras Dermatol.

[30] Sehgal V.N., Jain M.K., Srivastava G. (1989). Changing pattern of cutaneous tuberculosis. A prospective study. Int J Dermatol.

[31] Mann D., Sant’Anna F.M., Schmaltz C.A.S., Rolla V., Freitas D.F.S., Lyra M.R. (2019). Cutaneous tuberculosis in Rio de Janeiro, Brazil: description of a series of 75 cases. Int J Dermatol.

[32] Terranova M., Padovese V., Fornari U., Morrone A. (2008). Clinical and epidemiological study of cutaneous tuberculosis in northern Ethiopia. Dermatology.

[33] Azevedo T.P., Oliveira M.L. (2016). Analysis of cutaneous tuberculosis cases reported from 2000 to 2013 at a university hospital in Rio de Janeiro. Rev Soc Bras Med Trop.

[34] Aggarwal P., Singal A., Bhattacharya S.N., Mishra K. (2008). Comparison of the radiometric BACTEC 460 TB culture system and Löwenstein–Jensen medium for the isolation of mycobacteria in cutaneous tuberculosis and their drug susceptibility pattern. Int J Dermatol.

[35] (2000). Diagnostic standards and classification of tuberculosis in adults and children: this official statement of the American thoracic society and the centers for disease control and prevention was adopted by the ATS board of directors, July 1999. This statement was endorsed by the council of the infectious disease society of America, September 1999. Am J Respir Crit Care Med.

[36] Batista M., Ferreira B., Cruz G., Figueiredo A. (2019). Scrofuloderma: a diagnosis to bear in mind in the western world. Acta Med Port.

[37] Solis A.H., González N.E.H., Cazarez F., Pérez P.M., Olivera Diaz H.O., Escobar-Gutierrez A. (2012). Skin biopsy: a pillar in the identification of cutaneous Mycobacterium tuberculosis infection. J Infect Dev Ctries.

[38] Ramam M., Malhotra A., Tejasvi T., Manchanda Y., Sharma S., Mittal R. (2011). How useful is the Mantoux test in the diagnosis of doubtful cases of cutaneous tuberculosis?. Int J Dermatol.

[39] Verma P., Singal A. (2014). Utility of Mantoux test in the diagnosis of doubtful cases of cutaneous tuberculosis. Int J Dermatol.

[40] Siqueira R.C., Oréfice F. (2019). The potential of the IGRA (Interferon Gamma Release Assay) test for the diagnosis of ocular tuberculosis. Review and comparative analysis with the tuberculosis skin test. Rev Bras Oftalmol.

